# A Colônia Juliano Moreira de Bispo do Rosário revivida na obra de Gilmar Ferreira

**DOI:** 10.1590/S0104-59702025000100019

**Published:** 2025-05-23

**Authors:** Andrea Maia Gonçalves Pires, Priscila Faulhaber

**Affiliations:** i Doutoranda, Pós-Graduação em Museologia e Patrimônio/Universidade Federal do Estado do Rio de Janeiro. Rio de Janeiro – RJ – Brasil. andreamaiamg@gmail.com; ii Pesquisadora titular, Museu de Astronomia e Ciências Afins; professora, Pós-Graduação em Museologia e Patrimônio/Universidade Federal do Estado do Rio de Janeiro. Rio de Janeiro – RJ – Brasil. priscila@mast.br

**Keywords:** Colônia Juliano Moreira, Saúde mental, Museu, Arte, Brasil, Juliano Moreira Colony, Mental health, Museum, Art, Brazil

## Abstract

O estudo analisa o processo de produção artística de usuários da saúde mental na Colônia Juliano Moreira, considerando as mudanças no tratamento da loucura. Com base em pesquisa documental e na análise das obras de arte, o estudo evidencia a interseção entre a arte, a saúde mental e as transformações do ambiente de confinamento em espaço de expressão a fim de refletir sobre o poder disciplinar. Destacam-se a internação de Arthur Bispo do Rosário, em 1939, a fundação do Museu Bispo do Rosário Arte Contemporânea, na década de 1980, e a criação do Ateliê Gaia, em 2003.

## Abstract (en)

This study analyzes the art production process of mental health users at the Juliano Moreira Colony (Colônia Juliano Moreira) in light of changes in the treatment of insanity. Drawing on documentary research and analyses of artworks, the study focuses on intersections between art, mental health, and the transformations of a place of confinement into a space for expression in its reflections on disciplinary power. Milestones of note are the hospitalization of Arthur Bispo do Rosário in 1939, the founding of the Bispo do Rosário Contemporary Art Museum in the 1980s, and the creation of the Ateliê Gaia studio in 2003.

Este estudo propõe correlacionar as produções artísticas de dois pacientes que estiveram sob cuidados em uma instituição psiquiátrica em diferentes períodos da história do tratamento da saúde mental: no final do século XX e nas primeiras décadas do século XXI. Buscamos contribuir para reflexões sobre as mudanças nos saberes relativos aos portadores de sofrimento psíquico, classificado como loucura, e nas práticas terapêuticas com o uso da arte. Examinamos aspectos da história da instituição Colônia Juliano Moreira (CJM), bem como a gênese do Museu Bispo do Rosário Arte Contemporânea (MBRAC) e os seus desdobramentos.

Examinou-se, primeiramente, a produção de Arthur Bispo do Rosário (1909 ou 1911-1989),^1^ artista mundialmente conhecido que viveu na CJM. Sua vida e sua obra foram profundamente influenciadas pelo controle disciplinar (Foucault, 1999). Arthur Bispo do Rosário criou a maioria de suas produções artísticas durante o período do modelo asilar, caracterizado por exclusão social e isolamento dos pacientes. Essa prática se fundamentava na premissa do alienismo e no seu “tratamento moral”, que considerava o isolamento uma medida terapêutica essencial. Durante sua internação, Bispo enfrentou violência psiquiátrica e aniquilação da personalidade, comuns em manicômios da época (Lougon, 1993; Venancio, 2011; Araújo, Fernandes, 2021).

Estabelecem-se correlações com as atividades artísticas de um usuário^2^ contemporâneo, Gilmar Ferreira (1966-), que iniciou seu tratamento na CJM em 1986, com condutas terapêuticas diferentes das vivenciadas por Bispo. O período coincide com o surgimento da reforma psiquiátrica no Brasil, iniciada pelo Movimento dos Trabalhadores em Saúde Mental (MTSM) em 1978, durante o processo de redemocratização do país. A reforma propôs a ruptura com a lógica manicomial e a transição para a atenção psicossocial, promovendo iniciativas de inclusão social dos pacientes. Nesse processo, críticas ao modelo psiquiátrico clássico enfatizaram a construção de um novo lugar social para a loucura e incentivaram ações para integração desses pacientes na sociedade (Amarante, 1995; Amarante, Nunes, 2018; Delgado, 1992; Machado et al., 1978; Tenório, 2002).

A pesquisa explora e compara os contextos cultural e científico dos artistas Bispo e Ferreira, analisando como suas influências estéticas e os procedimentos criativos refletem os diferentes momentos históricos em que viveram. Ao abordar a produção artística em manicômios fechados e após a reforma psiquiátrica, destaca-se a importância das mudanças nos modelos de tratamento e na percepção e valorização da arte. Bispo e Ferreira, ambos usuários da CJM, representam contextos históricos distintos que influenciaram suas produções artísticas e suas compreensões sobre a arte. A análise também contextualiza observações em debate com outros autores, comparando períodos, estéticas, procedimentos e resultados. O objetivo é entender como essas mudanças históricas e culturais impactaram as vivências e produções artísticas de Bispo e Ferreira.

Durante o período entre as duas guerras mundiais, com a disseminação da psicanálise, do modernismo e das vanguardas artísticas no Brasil, a arte produzida nos asilos e seus criadores passaram a ser vistos sob uma nova perspectiva, promovendo mudanças significativas na psiquiatria. Nas décadas de 1920 e 1940, pioneiros como Osório Thaumaturgo Cesar (1895-1979) e Nise da Silveira (1905-1999) trabalharam com terapia ocupacional no tratamento psiquiátrico, vendo a arte manicomial como material terapêutico e estético. Esse período foi marcado pela circulação de ideias que destacavam a importância da arte dos “insanos” no cenário artístico e científico, integrando obras de pacientes em exposições de arte moderna. Figuras como Osório Cesar defendiam a valorização estética e terapêutica dessas produções, alterando a percepção das obras e impulsionando mudanças nas práticas psiquiátricas, incorporando a arte como ferramenta terapêutica amplamente reconhecida e valorizada (Facchinetti, 2023).

Cabañas (2018b) propôs uma abordagem teórica e histórica para a arte criada em diálogo com o trabalho de pacientes psiquiátricos. O trabalho artístico desses pacientes, longe de ser marginal às instituições da arte moderna no Brasil, foi tratado como arte e exibido em espaços artísticos desde pelo menos 1933. Assim, a história do trabalho desses pacientes é parte constitutiva do modernismo, e não algo externo a ele. A autora explora as convergências entre o modernismo e a sua relação com a psiquiatria no Brasil. Enquanto na Europa filósofos e artistas modernistas mostravam-se intrigados pela loucura, no Brasil emergiu a “arte dos insanos”, que floresceu articulada aos movimentos modernistas, especialmente entre as décadas de 1920 e 1960. A criação de arte pelos pacientes psiquiátricos, incentivada por figuras proeminentes na medicina e na crítica de arte, levou à valorização positiva dessas obras pelos curadores das principais instituições de arte moderna no Brasil, tendo sido incluídas em importantes exposições e coleções.

A escassez de pesquisas que documentem as produções artísticas dos usuários do serviço de saúde mental da CJM motiva o presente trabalho. O estudo destaca a importância da produção artística dos usuários para a formação de coleções museológicas na arte contemporânea brasileira e mundial, evidenciada pela participação de suas obras em bienais de arte e outras exposições nacionais e internacionais, como a exposição individual “Bispo do Rosario: all existing materials on Earth” na Americas Society em Nova York, em 2023.

## Instituições totais, poder disciplinar e produção artística em manicômios

Diferentes análises oferecem perspectivas variadas para a compreensão das dinâmicas sociais e do funcionamento das instituições de controle e poder. Entre essas abordagens, destaca-se a definição de instituição total como “um local de residência e de trabalho onde um grande número de indivíduos com situação semelhante, separados da sociedade mais ampla por um período considerável de tempo, levam uma vida fechada e formalmente administrada” (Goffman, 2015, p.11). Segundo esse autor, essas instituições, como sanatórios, hospitais psiquiátricos e leprosários, são estabelecidas para o cuidado de pessoas consideradas incapazes de cuidar de si mesmas e que representam uma ameaça não intencional para a comunidade. No bairro de Jacarepaguá, no Rio de Janeiro, além da CJM, há outras instituições que se enquadram nessa classificação, como o Hospital Curupaiti, fundado em 1929, para cuidar principalmente de pessoas atingidas pela hanseníase,^3^ e o Hospital Raphael de Paula Souza, inaugurado em 1952, para tratar pacientes com tuberculose.

Michel Foucault examinou o exercício do poder em prisões, escolas e manicômios. Em sua obra *Vigiar e punir: nascimento da prisão* (Foucault, 1999), explorou a história e a evolução dos sistemas de punição na sociedade ocidental, desde a punição física até o surgimento das instituições disciplinares, com foco na prisão. Ele criticou o “humanismo” dos reformadores penais do século XVIII, destacando a transição de um modelo baseado no sistema punitivo para um poder disciplinar calcado em técnicas de vigilância, com base na “economia política do corpo”, que garantia a submissão dos corpos individualizados (Foucault, 1999, p.25).

Segundo Foucault (1999), a sociedade disciplinar constituiu instituições que desempenham um papel central na vigilância, na normatização e no constante exame dos indivíduos, deixando marcas nos corpos e impondo condutas. As práticas disciplinares regulam o exercício do controle normativo e produzem conhecimento. De maneira sutil, nem sempre coercitiva, essas práticas moldam mentes e corpos dóceis: “É dócil um corpo que pode ser submetido, que pode ser utilizado, que pode ser transformado e aperfeiçoado” (p.118). Corpos disciplinados são ativos valorizados pela sociedade, atendem às diversas intenções do poder por meio de técnicas meticulosas de controle, observação e punição.

Durante a Idade Clássica, o corpo era tratado como objeto e alvo de poder, evidenciado pelo interesse em manipulá-lo, modelá-lo e treiná-lo, revelando uma dualidade entre sua utilidade e sua inteligibilidade. Foucault (1999) argumentou que as disciplinas, métodos de controle minucioso que impõem uma relação de docilidade-utilidade ao corpo, surgiram como formas gerais de dominação nos séculos XVII e XVIII. Essas disciplinas constituem uma arte do corpo humano, ao formar uma relação que torne o corpo tanto mais obediente quanto mais útil, dando origem a uma “anatomia política” e a uma “mecânica do poder”. A disciplina fabrica corpos submissos e exercitados, enquanto aumenta suas forças em termos de utilidade econômica e diminui em termos de obediência política (Foucault, 1999).

Ao afirmar que “a disciplina é uma anatomia política do detalhe”, Foucault (1999, p.120) exemplificou que a minúcia dos regulamentos, a vigilância atenta das inspeções e o controle sobre os aspectos mínimos da vida e do corpo aplicados na escola, no quartel, no hospital ou na oficina conferem um conteúdo secularizado e uma racionalidade econômica ou técnica a essa atenção minuciosa ao ínfimo e ao infinito.

Durante o período em que Bispo foi paciente na CJM, de 1939 a 1989, a instituição utilizava práticas disciplinares rigorosas, com vigilância constante e controle estrito do comportamento dos pacientes. Essas normas tinham como objetivo moldar as ações e os pensamentos dos indivíduos. No entanto, essas instituições também geravam conflitos e contradiscursos. A produção artística de Bispo, por exemplo, expressava sua singularidade e reagia ao controle e à normalização impostos pelo manicômio.

Foucault, em um curso ministrado no Collège de France, anos após *Vigiar e punir*, explorou um tema bastante diferente das práticas disciplinares, voltando-se para a hermenêutica do sujeito, o que ele chama de “cuidado de si”, relacionado às formas de subjetivação (Foucault, 2006). Apesar das abordagens distintas, cabe considerar as convergências na análise dos sujeitos artistas inseridos em instituições de tratamento da saúde mental. Mesmo que em momentos e práticas diferentes da história desses tratamentos, é importante considerar as circunstâncias em que tais sistemas de subjetivação constituíram esses indivíduos como artistas e, assim, examinar como eles conceberam aspectos de suas obras.

Nos seus estudos finais, Foucault (2006) revisitou a Antiguidade Clássica para reconsiderar a ética moderna por meio de uma ontologia crítica do presente. Ele demonstrou que o processo de subjetivação do indivíduo clássico, ao adotar uma posição ética, direcionava-se para uma estética da vida. Em *A hermenêutica do sujeito* (2006), Foucault detalha as “práticas de si”, as “técnicas da existência” e o “cuidado de si” como elementos centrais na construção de um modo de vida baseado em escolhas pessoais que criam um estilo de vida como “obra de arte”. Essa ética, extraída dos antigos, é uma verdadeira estética da existência, uma liberdade possível no processo de fazer-se existir.

As principais ideias do curso de Foucault sobre a hermenêutica de si destacam a importância do cuidado de si como prática filosófica, social e terapêutica na Antiguidade. A análise foucaultiana do cuidado de si, presente em *A hermenêutica do sujeito* (2006), é importante para compreender a produção artística de Bispo e o seu contexto de vida na CJM. Foucault enfatiza que, na Antiguidade, o cuidado de si envolvia práticas que iam além da mera introspecção, englobando técnicas e exercícios que permitiam ao indivíduo sofrer adversidades e moldar sua subjetividade. Esse conceito é particularmente relevante para entender como Bispo, mesmo sob condições extremas de confinamento e abuso psiquiátrico, conseguiu criar uma obra de grande valor artístico e expressivo.

A produção artística de Bispo, portanto, pode ser vista como uma forma de “cuidado de si”, transformando sua experiência de isolamento em uma prática de autoconhecimento. Sua obra reflete as condições adversas em que foi criada e demonstra como a arte pode servir como uma ferramenta de subversão diante do poder disciplinar.

Por meio de sua produção artística, Bispo subvertia o poder disciplinar, criando um ateliê em seu “quarto-forte” – um espaço de autonomia que contrastava com as tentativas da instituição de moldar e controlar sua subjetividade. Suas obras, muitas vezes compostas por objetos cotidianos transformados em arte, simbolizavam uma rejeição à ordem estabelecida e uma afirmação de sua identidade como criador. Esse processo criativo funcionava como um meio de lidar com as imposições do manicômio, ao mesmo tempo que criava um discurso voltado para o mundo exterior. Entende-se que a arte de Bispo criou significados em um ambiente de opressão, ao inserir a loucura no discurso e utilizar-se de sua condição para reinventar o sistema.

## A criação da Colônia Juliano Moreira e as produções artísticas

No Brasil do século XIX, a psiquiatria se estabeleceu como um campo da medicina social, e os hospícios foram criados com propósitos terapêuticos psiquiátricos, em resposta à necessidade de controlar a desordem urbana e a população desviante (Machado et al., 1978). Entre 1852 e 1902, a produção artística dos alienados no Hospício de Pedro II serviu para recreação, diagnóstico e estudos científicos, mas não era valorizada ou divulgada, sendo frequentemente ignorada. A racionalidade moderna focava o tratamento das doenças mentais, negligenciando o valor terapêutico e expressivo da arte. A arte dos pacientes fornecia aos alienistas indícios sobre seus estados mentais, auxiliando no diagnóstico e no desenvolvimento de teorias sobre a loucura, mas era vista sob uma lente médica e científica, com pouca consideração pelo valor artístico (Facchinetti, 2022).

O início do século XX foi marcado, no país, por mudanças significativas e investimentos voltados para a assistência a indivíduos marginalizados, como parte das medidas de saúde pública implementadas durante o mandato do prefeito Francisco Pereira Passos (1902-1906) e do diretor-geral de Saúde Pública, Oswaldo Cruz, no governo de Rodrigues Alves. A promulgação do decreto n.1.132, em 22 de dezembro de 1903, que reformulou os sistemas de apoio a esses indivíduos, juntamente com a nomeação de Juliano Moreira^4^ como chefe do Hospício Nacional no mesmo ano, resultou em reformas estruturais e de bem-estar. Essas ações aprimoraram os esforços modernizadores do governo e expandiram a assistência pública aos marginalizados (Venancio, 2011).

Em 1912, a Fazenda do Engenho Novo, no Rio de Janeiro, foi desapropriada para a criação da Colônia de Alienados de Jacarepaguá, devido à sua extensa área, composta por matas fechadas, rios e cachoeiras. A instituição foi inaugurada em 1924, e, em outubro de 1935, passou a se chamar Colônia Juliano Moreira, em homenagem ao doutor Juliano Moreira, figura central na institucionalização da assistência e ciência psiquiátrica no Brasil (Venancio, 2022).

Em fins dos anos 1930 e principalmente início da década de 1940, a CJM passou por acentuado processo de expansão de sua estrutura física e de seus recursos terapêuticos. No contexto das políticas públicas para a psiquiatria forjadas a partir do final da década de 1930 e concretizadas em boa medida na década seguinte, a colônia foi sendo transformada em um hospital-colônia (Venancio, 2011).

Na década de 1940, a CJM utilizava diversos recursos terapêuticos, como assistência heterofamiliar, praxiterapia, convulsoterapia (elétrica e química), choque insulínico, eletronarcose e psicocirurgia. As principais atividades da praxiterapia incluíam lavoura de cereais e hortaliças, pecuária e pequenas indústrias, como a produção de artefatos de vime e colchões, empregando cerca de 1.600 doentes (Venancio, Cassilia, 2007). Os pacientes também participavam de atividades recreativas e culturais, como pintura, música, rádio, cinema, teatro e esportes (Peres, 1949).

A infraestrutura da CJM era composta por pavilhões separados por gênero, muitas vezes em condições precárias, com superlotação, higiene inadequada e cuidados médicos limitados. De 1920 a 1980, a colônia abrigou cerca de cinco mil pacientes considerados irrecuperáveis. Bispo esteve internado intermitentemente de 1930 a 1980, período em que produziu grande parte de suas obras. As práticas médicas visavam segregar os doentes da sociedade para protegê-la e tratar os pacientes em ambientes isolados, refletindo uma política de confinamento e controle.

## Entre manicômios e reformas: investigando estéticas e terapêuticas nas criações artísticas de Bispo e de Ferreira

Este estudo empregou uma abordagem qualitativa, integrou pesquisa teórica e exploratória, incluiu revisão da literatura e entrevistas semiestruturadas. Destacaram-se as trajetórias de Bispo e de Ferreira, bem como as suas produções artísticas, considerando a influência do ambiente manicomial nas obras de Bispo e de Ferreira e como as mudanças institucionais afetaram a estética e os métodos de criação artística dos usuários de saúde mental. Foram analisados momentos específicos nas trajetórias de ambos, como a internação, a abordagem terapêutica, a produção artística e o reconhecimento da obra. Essas análises visaram destacar as diferentes perspectivas e visões de mundo dos dois distintos períodos históricos em que esses usuários viveram na CJM.

A história de vida de Bispo e a sua coleção estão interligadas à trajetória da CJM e do MBRAC; enquanto a história de vida de Ferreira está associada às mudanças na CJM, no MBRAC e ao surgimento do Ateliê Gaia. Compararam-se modelos de tratamento, estéticas e procedimentos em dois momentos históricos da CJM, o que permite discutir a questão dos usos da arte produzida por Bispo em manicômios fechados e aquela produzida por Ferreira após a reforma psiquiátrica.

A pesquisa documental concentrou-se nos arquivos da CJM, no acervo do MBRAC e nos registros de prontuários dos pacientes arquivados no Instituto Municipal de Assistência à Saúde Juliano Moreira (IMAS JM), todos sob a responsabilidade da Secretaria Municipal de Saúde do Rio de Janeiro. Privilegiaram-se fontes primárias, como textos psiquiátricos da época, para compreender a rotina do hospital, incluindo seus procedimentos de diagnóstico e terapêutica, dentro de seu contexto histórico e cultural.

## O artista morador de pavilhão na Colônia Juliano Moreira

Nascido provavelmente em 1909, em Japaratuba, Sergipe, Arthur Bispo do Rosário era filho de um carpinteiro. Homem negro, nordestino e pobre, Bispo ingressou na Escola de Aprendizes Marinheiros em Aracaju em 1925. No ano seguinte, mudou-se para o Rio de Janeiro, onde se alistou na Marinha de Guerra e permaneceu por nove anos, período em que conheceu o boxe. Após deixar a Marinha, trabalhou na empresa Light and Power, de 1933 a 1937, enquanto investia em sua carreira como pugilista. Em 1936, sofreu um acidente em que seu pé direito foi esmagado pela roda de um bonde, deixando-o manco. Durante o processo trabalhista contra a Light and Power, Bispo conheceu Humberto Leone, advogado da sua causa, e passou a residir e trabalhar na casa deste, em Botafogo, no Rio de Janeiro (Hidalgo, 2011).

Em 22 de dezembro de 1938, Bispo teve uma revelação que mudaria sua vida: ele se viu descendo do céu, acompanhado por sete anjos, que o deixaram na “casa nos fundos murados de Botafogo”, conforme relatado em um de seus estandartes bordados. Naquela noite, Bispo vagou pelas ruas até chegar ao Mosteiro de São Bento, no Centro do Rio. Foi levado ao antigo Hospital Nacional dos Alienados, na Praia Vermelha, pela autoridade policial, sob a alegação de indigência, e transferido para a CJM no início de 1939. Após triagem, foi alojado no pavilhão 11 do Núcleo Ulisses Vianna (Hidalgo, 2011).

Segundo as fichas em seu prontuário, incompletas devido à falta de páginas e à escassez de informações, Bispo foi internado na CJM em janeiro de 1939, aos 27 anos, classificado como indigente e solteiro, sem informações sobre o ano de nascimento, filiação ou profissão (Prontuário, jan. 1939). Entre 1940 e 1960, Bispo alternou períodos de internação na CJM com momentos em que exercia algum ofício em residências cariocas. Em 1964 regressou à CJM, onde permaneceu internado até sua morte, em 5 de julho de 1989, com diagnóstico de esquizofrenia paranoide (Hidalgo, 2011).

A trajetória de Bispo durante sua produção artística na CJM não foi favorável ao seu desenvolvimento como artista. Bispo enfrentou várias dificuldades na área social e em relação à sua saúde. Não recebeu treinamento técnico, não conviveu com outros artistas nem leu livros especializados. Também não frequentou espaços de arte e não tinha um local apropriado para suas criações. Todas essas dificuldades, contudo, não inviabilizaram sua expressão artística (Ferrão, 2014).

## Investigação dos modelos médicos e compreensão sobre arte

O modelo manicomial, surgido no final do século XVIII, baseava-se no isolamento terapêutico, no afastamento das pessoas com doença mental do convívio social e familiar. A inauguração do asilo por Philippe Pinel (1745-1826), em 1793, marcou o início do alienismo, fundamentado na reclusão para conhecer e tratar a loucura. O “isolamento terapêutico” era central na tecnologia pineliana, ao associar a loucura ao asilo como medida de proteção tanto para o indivíduo quanto para a sociedade. Esse duplo processo de “isolar para saber” e “isolar para tratar” consolidou-se nas práticas de saúde mental (Amarante, Torre, 2018). O modelo asilar, baseado na psiquiatria clássica, enfatizava a institucionalização dos pacientes, mantendo-os segregados e isolados da sociedade, negando-lhes o direito à participação social e à cidadania plena (Amarante, 1998).

O relatório médico de 8 de fevereiro de 1985 descreve a rotina de Bispo, que consistia principalmente em permanecer recluso, dedicando-se a atividades manuais que ele mesmo inventava. Embora notavelmente singular em seu isolamento, ele não era o único paciente envolvido em atividades criativas. Na CJM, existia um programa abrangente de praxiterapia, que incluía oficinas de costura e bordado (Cabañas, 2018a).

Entre 1950 e 1951, apenas cinco internos da CJM participaram de oficinas artísticas, número insignificante comparado ao das demais atividades de trabalho braçal na colônia. No entanto, essa iniciativa contribuiu para melhorar a percepção pública do funcionamento da instituição, representou um avanço na reabilitação e era valorizada por alguns médicos e artistas (Araújo, Jacó-Vilela, 2018).

Nos primórdios dos anos 1980, em consonância com os ideais da reforma psiquiátrica, os tratamentos com eletrochoque foram suspensos na CJM. Isso incluía a eletroconvulsoterapia, intervenção temida pelos internos que, além da suposta justificativa médica, era utilizada com propósitos disciplinares, direcionada àqueles que infringiam as normas de conduta. Outra mudança significativa ocorreu a partir da década de 1980, últimos anos da vida de Bispo: os “quartos-fortes” do Núcleo Ulisses Vianna foram desativados. Eram celas individuais, equipadas com catre e latrina de cimento, fechadas por portas gradeadas de ferro ou madeira espessa. Além de desativadas, elas tiveram algumas paredes derrubadas para criar mais espaço de armazenamento para as obras de Bispo (Araújo, 2016). A iniciativa de abrir tais locais de reclusão simbolizou um momento de grande importância, marcando a transição entre a “colônia velha”, autoritária e isolada, e a “colônia nova”, democrática e acessível (Lougon, 1993, p.145).

Bispo recebeu reconhecimento por sua produção artística ainda em vida, destacando-se na história da arte contemporânea e na história da psiquiatria no Brasil. Os primeiros anos de reconhecimento de sua obra ocorreram por meio de diversas fontes (O prisioneiro..., 1982; O Bispo, 1985; Rosário, 1988). Em 1982, ocorreu a primeira exposição dos seus estandartes na mostra “Margem da vida”, no Museu de Arte Moderna do Rio de Janeiro. Esses registros, juntamente com seus escritos e bordados, oferecem um relato de sua vida e obra.

## Entre paredes: a produção artística de Bispo na Colônia Juliano Moreira

As obras de Bispo refletem seu pensamento e suas vivências na atmosfera do manicômio no qual esteve internado, cercado por objetos do contexto hospitalar, bem como por itens doados pelos funcionários e amigos. Muitos desses objetos foram minuciosamente coletados pelo artista nas instalações e nos pátios do hospital psiquiátrico e, posteriormente, incorporados às suas criações. Esses itens não apenas testemunham a história da instituição, mas também narram a trajetória pessoal de Bispo e as suas interações ao longo da vida. Documentos do Instituto do Patrimônio Histórico e Artístico Nacional revelam que a produção artística de Bispo inclui elementos como nomes de pessoas, lugares, datas, números, paisagens, cosmovisões e objetos que conectam seu universo interior e exterior, juntamente com uma riqueza de símbolos e signos a serem decifrados (Brasil, 2018, p.36).

Conhecido como “xerife”, Bispo ganhou essa fama por auxiliar os guardas do hospital a conterem outros pacientes em momentos de crise. Essa capacidade de Bispo se deve aos treinos de força realizados durante sua preparação para se tornar pugilista. Sua identificação e a colaboração com os guardas proporcionaram a ele certos privilégios na hierarquia do manicômio, além de proteger sua coleção artística. Suas obras não eram fruto de qualquer indicação de praxiterapia, como era chamada a terapêutica ocupacional na CJM (Hidalgo, 2011; Araújo, 2016). Segundo os documentos do Instituto Estadual do Patrimônio Cultural do Rio de Janeiro, Bispo produziu 802 obras, que compõem o acervo do MBRAC, incluindo panos, estandartes, *assemblages*,^5^ mantos múltiplos, faixas e outros objetos (Rio de Janeiro, 1994).

Parte dessas obras pode ser considerada um registro tridimensional da vida cotidiana desse paciente no manicômio. Bispo acumulou talheres, canecas, pedaços de madeira, arame, vassoura, papelão, fios de varal, garrafas, botões, pentes, sapatos, peças de roupas, uniformes de pacientes que, após desfiados, forneceram ao artista a linha azul usada para seus bordados. Em seu confinamento, bordou a escrita de seus estandartes e fragmentos de tecido. Em seu delírio místico, imbuído da suposta missão de reconstruir o mundo para se apresentar a Deus no dia do Juízo Final, produziu milhares de objetos que formaram uma coleção (Hidalgo, 2011; Morais, 2013). Suas produções artísticas não foram criadas com a intenção de serem comercializadas no mercado de arte. Em vez disso, seus objetos eram uma representação das coisas do mundo, destinada a Deus, já que atribuía um valor sagrado à sua obra (Wellisch, 2006).

Destaca-se o *Manto da apresentação*, peça que sintetizou as criações de Bispo e foi exibida em museus brasileiros e no exterior. Morais (2013, p.97) o considerou sua obra-prima, “a síntese mental e visual” de sua obra. Ele criou essa vestimenta a partir de um cobertor marrom do manicômio que foi meticulosamente bordado em ambas as faces, decorado com diversas cordas coloridas que cruzam da frente às costas do manto e terminam em borlas decorativas e franjas. Na parte externa, bordou imagens e textos que representam seu universo, enquanto, na parte interna, nomes de pessoas queridas. Esses nomes foram escritos, em sua maioria, com um fio azul sobre tecido branco.

Bispo produziu seu manto para ser usado no momento de sua morte e em sua chegada aos céus. Como preparação para sua apresentação a Deus: ”Momento tão esperado por Bispo para que a comunicação entre céu e terra acontecesse plenamente, possibilitando a entrega do mundo em miniaturas a seu Pai e o início do Julgamento Final executado por Pai e Filho” (Figueiredo, 2010, p.93). Em suas próprias palavras: “Quando eu subir, os céus se abrirão e vai recomeçar a contagem do mundo. Vou nessa nave, com esse manto e essas miniaturas que representam a existência. Vou me apresentar… A minha morte se fará notar no mundo inteiro” (Silva, maio 2007, p.5).

Bispo colecionou e organizou objetos que formavam grupos tipológicos da vida cotidiana, as “vitrines”, como se desejasse mostrar a alguém desconhecido o que usamos para beber, vestir, comer, construir e celebrar. Além disso, suas *assemblages*, como denominadas pelo historiador e crítico de arte Frederico Morais, indicam a reunião de objetos acumulados e vão além de simples colagens.

Na obra *Congas e Havaianas*, o artista apresentou os sapatos Congas e os chinelos Havaianas, usados como parte do uniforme hospitalar dos pacientes na CJM, amarrados em um suporte de madeira. Juntamente com as obras *Caneca* e *Talheres*, que representam canecas e talheres utilizados pelos pacientes, compuseram a exposição na Bienal de Veneza de 1995, na qual Bispo representou o Brasil.

A *Roda da fortuna* de Bispo frequentemente provoca debates entre os pesquisadores de história da arte devido à sua semelhança visual com a *Roda de bicicleta*, famoso *ready-made* de Marcel Duchamp. No entanto, as semelhanças são principalmente visuais, pois as motivações e intenções dos dois artistas, Bispo e Duchamp, são substancialmente distintas. Enquanto Bispo era impulsionado pela fé, Duchamp contestava valores estabelecidos culturalmente, antecipando a arte conceitual (Boppré, 2013). A obra de Bispo, *Roda da fortuna*, é uma montagem escultórica em ferro, madeira e saco plástico. Já a obra de Duchamp, *Roda de bicicleta*, de 1913, consiste em uma roda de bicicleta montada sobre um banquinho. *Roda de bicicleta* marcou o início da proposta *ready-made*, na qual objetos prontos, não criados pelo artista, são incorporados à produção artística, uma abordagem que ganhou destaque a partir da década de 1960 (Girst, 2003).

## A fundação do Museu Bispo do Rosário Arte Contemporânea e do Ateliê Gaia

O MBRAC teve suas origens na década de 1950, quando foi estabelecido um departamento na CJM para abrigar produções artísticas, que acabou sendo o embrião do museu. Na década de 1980, foi fundado ali o Museu Nise da Silveira, em homenagem à psiquiatra que destacou o conteúdo a ser revelado nas obras dos pacientes, embora ela nunca tenha trabalhado na CJM.

Após o falecimento de Bispo, em 1989, o reconhecimento de suas produções artísticas como obras de arte, juntamente com sua participação em exposições, e diante dos avanços da reforma psiquiátrica no Brasil, a equipe da CJM determinou o destino das obras produzidas por Bispo ao longo dos cinquenta anos em que esteve internado intermitentemente. Foi iniciado, então, pela Associação dos Amigos dos Artistas da Colônia Juliano Moreira, um processo de deslocamento dessa coleção do Núcleo Ulisses Vianna para o Museu Nise da Silveira (Araújo, 2016).

A associação, além de preservar a coleção de Bispo e os direitos dos artistas da CJM, organizou exposições, atividades culturais, eventos, pesquisas, palestras, debates e outros meios de educação e informações audiovisuais relacionados à arte e à saúde mental. Financiou também uma oficina de artes nas instalações do museu que se tornou a semente para o futuro Ateliê Gaia (Fabrício et al., 2016).

**Figura 1:**
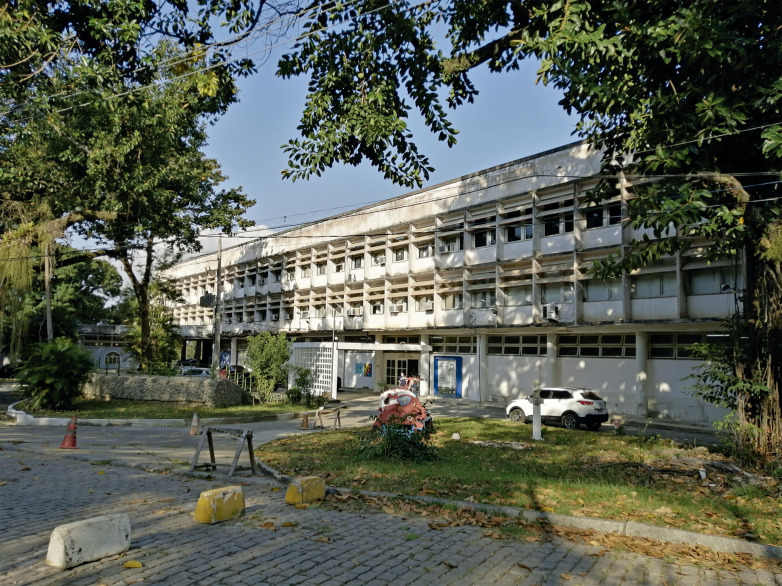
: Fachada principal da sede administrativa do IMAS JM, Edifício Heitor Peres, onde funciona o Museu Bispo do Rosário Arte Contemporânea (foto feita por Andrea Pires, 2022)

Na década de 2000, o psiquiatra Ricardo Aquino assumiu a gestão do museu e buscou uma visão inovadora, explorando a ideia de um “museu da criação”, imprimindo nova orientação teórica para a instituição. O então diretor, seguindo essa visão, alterou o nome do museu duas vezes: primeiro para Museu Bispo do Rosário e, posteriormente, acrescentando “arte contemporânea”, o que resultou no nome atual, Museu Bispo do Rosário Arte Contemporânea (Figura 1), que incorpora a missão autoatribuída pelo célebre paciente, ressignificando o próprio mundo. Aquino (2004, 2010), em contraposição à ideia de museu do acúmulo de poeira, incentivou a criação agenciada por artistas, e não por técnicos da saúde mental, desmedicalizando e “despsiquiatrizando” a criação artística, a fim de promover a cidadania e a diversidade.

O Ateliê Terapêutico Ocupacional Gaia foi criado em 2003, separado do museu, por haver divergências nos objetivos da condução do tratamento de saúde mental e na orientação das práticas das produções artísticas entre o diretor do museu e a terapeuta ocupacional Rita de Cássia Barcellos Bittencourt, coordenadora do ateliê (Fabrício et al., 2016). Submetido diretamente ao IMAS JM, o serviço do Ateliê Gaia concentrou-se em oferecer atividades expressivas por meio de espaços singularizados de atenção, no qual a arte era considerada uma ferramenta de ação fundamental (Soares, 2007). Alinhado mais estreitamente à área de conhecimento da terapia ocupacional, o Ateliê Gaia representou um espaço significativo durante a transição do modelo asilar para a atenção psicossocial.

Em 2013, a psiquiatra Raquel Fernandes assumiu a direção do museu e buscou maior conexão com a comunidade da Zona Oeste do Rio de Janeiro, apresentando a instituição como um dispositivo cultural regional e fomentando a integração local. Sob sua gestão, o MBRAC retomou a coordenação do Ateliê Gaia, enfocando a produção artística, a participação dos artistas e usuários em exposições e a venda de obras. Hoje, o MBRAC é vinculado ao IMAS JM e à Secretaria Municipal de Saúde do Rio de Janeiro e conta com uma equipe multidisciplinar que inclui profissionais das áreas de saúde mental, museólogos, curadores, produtores, artistas e educadores (Araújo, Fernandes, 2021).

## Gilmar Ferreira: “o artista que provoca, discute e aponta caminhos”6

Gilmar Ferreira se apresenta em suas exposições como artista plástico e cidadão ativo na comunidade da Cidade de Deus, no Rio de Janeiro. Ele não hesita em revelar sua condição de paciente assistido por uma instituição psiquiátrica: “Luto através de minha arte contra preconceitos e estigmas que ampliam o lado negativo da pobreza, marginalidade, violência psiquiátrica, negritude e abandono” (Ferreira, out. 2008).

O prontuário de Gilmar na antiga CJM registra as seguintes informações: Gilmar Ferreira da Silva, nascido em 9 de setembro de 1966, natural de Campo Grande, Rio de Janeiro, pardo, sexo masculino e casado. Filho de Severino Ferreira da Silva e Alais dos Santos Silva, que residem na Cidade de Deus, Rio de Janeiro. Sua primeira internação foi em 3 de março de 1986, quando tinha 20 anos (Prontuário…, 3 mar. 1986).

Enquanto no período em que Bispo viveu na instituição o isolamento era a tônica, na época do tratamento de Ferreira, a partir da década de 1980, o enfoque terapêutico se concentrou na interação com a sociedade – foram desativados os pavilhões de internação, transfigurando o lugar. Também foram formadas equipes multidisciplinares, compostas por médicos, psicólogos, terapeutas ocupacionais, museólogos, entre outros profissionais. Iniciou-se o processo de ressocialização dos pacientes de longa internação, com estratégias de reinserção destinadas a facilitar a reintegração dos pacientes na sociedade.

O Centro de Reabilitação e Integração Social foi criado como espaço destinado a acolher pacientes que não mais necessitavam de internação hospitalar, mas precisavam de reabilitação para o trabalho, a fim de serem efetivamente integrados à sociedade. Implementou-se o Bolsa Etapa, programa que oferece remuneração aos pacientes que participam de atividades laborativas dentro ou fora da colônia.

A concentração populacional e urbana na área da CJM e no seu entorno ocorreu em paralelo com as mudanças nos moldes da reforma psiquiátrica dos hospitais públicos federais do Rio de Janeiro. Essas transformações refletiram os esforços de reforma na área de saúde em geral, incluindo a criação do Sistema Único de Saúde (SUS) nos fins da década de 1980. Desde então, esses hospitais passaram por processos de municipalização. No caso da CJM, esse processo teve início em 1996, resultando na criação do IMAS JM, com o objetivo de fortalecer o SUS no âmbito psiquiátrico (Venancio et al., 2015). Com o crescimento populacional na área do IMAS JM, hoje temos um sub-bairro chamado Colônia, em Jacarepaguá. Anteriormente, havia um hospital-colônia caracterizável como instituição total, no sentido goffmaniano; na atualidade, no IMAS JM, temos um espaço de interação entre usuários de saúde mental e a comunidade.

No prontuário de Ferreira consta que suas internações foram realizadas por diferentes familiares, no Hospital Jurandyr Manfredini, uma das unidades da CJM. O hospital, criado nos anos 1980 como parte dos serviços da CJM, com o propósito de atender pacientes externos, busca interromper o fluxo de pacientes considerados irrecuperáveis, que vinham de todo o país para serem internados na instituição. Oferecia tratamento ambulatorial, internações de curta duração e serviços de atendimento de emergência psiquiátrica. Com a municipalização da CJM, o hospital também foi municipalizado e passou a se chamar Hospital Municipal Jurandyr Manfredini (HMJM). No entanto, encerrou suas atividades na década de 2020. Atualmente, após reformas, o local abriga o Centro de Atenção Psicossocial (Caps) Manoel de Barros, e, em 2022, lá foi inaugurada a Clínica da Família Arthur Bispo do Rosário.

Em 2011, Ferreira foi levado pelos familiares à emergência do HMJM em diversas ocasiões. O motivo alegado para sua internação seria que ele estava vestido com uma farda militar enquanto caminhava pelas ruas da Cidade de Deus, no Rio de Janeiro, abordando os policiais e afirmando fazer parte da milícia (Prontuário…, 3 mar. 1986). Frequentou as consultas no HMJM e passou por internações pontuais, com duração variável, em momentos de crise. Ao contrário de Bispo, Ferreira nunca residiu nos pavilhões da CJM e envolveu-se ativamente em terapias. Sua contínua produção de obras de arte, formação de coleções e participação em exposições no MBRAC e em outros museus são evidências claras de seu comprometimento com a expressão artística e de sua integração na comunidade artística. O engajamento de Ferreira no MBRAC e no Ateliê Gaia também ilustra as transformações ocorridas, incluindo a redução do número de espaços hospitalares e a emergência de locais de memória dentro desse contexto.

Em entrevista no restaurante Bistrô do Bispo, Ferreira mostrou uma pasta contendo diversos documentos pessoais, incluindo sua carteira de identidade e seu Cadastro de Pessoa Física. Além disso, exibiu um certificado de conclusão do ensino primário do Instituto Jesus, Maria, José, em Bento Ribeiro, estado da Guanabara, datado de 1959. Com orgulho, Gilmar compartilhou ainda sua experiência no Exército brasileiro. Esse é outro ponto em comum entre os dois artistas, tendo os dois servido nas forças armadas. Durante a entrevista, ele usava um chapéu com estampas semelhantes ao uniforme do Exército brasileiro, calça jeans que ele mesmo pintou e uma bota de cano longo preta. Além disso, mostrou um diploma de cidadania recebido devido à sua participação como voluntário na 15ª edição do “Fiocruz pra você”, uma campanha de vacinação realizada pela Fundação Oswaldo Cruz (Fiocruz) em 2008. Também exibiu um recibo de pagamento com a descrição “artista”, emitido pelo MBRAC por trabalhos realizados. Apresentou, ainda, sua carteira de matrícula no SUS, confirmando sua condição de usuário do Caps Manoel de Barros, localizado nas dependências da CJM, entre outros documentos (Ferreira, 2023).

A trajetória artística de Ferreira, conforme retratada nos catálogos e materiais de exposições, revela-o como um artista plástico autodidata que deu início à sua jornada nas artes na década de 1990. Convidado para monitorar a oficina de artes no antigo Museu Nise da Silveira, atualmente MBRAC, ele se tornou um dos primeiros artistas a desempenhar esse papel de forma remunerada. Suas primeiras exposições ocorreram no Museu Nacional de Belas Artes durante os anos 1990, incluindo uma individual em 1996 e uma exposição coletiva em 1998. A paixão pela pintura surgiu aos dez anos, enquanto auxiliava seu pai na pintura de carros em uma oficina, utilizando tintas remanescentes para criar obras em tábuas. Na década de 1990, Gilmar começou a experimentar desenhos e esculturas em barro e argila durante sua participação em oficinas terapêuticas na CJM. Na mesma década, realizou estudos na Escola de Artes Visuais do Parque Lage, no Rio de Janeiro.

**Figura 2:**
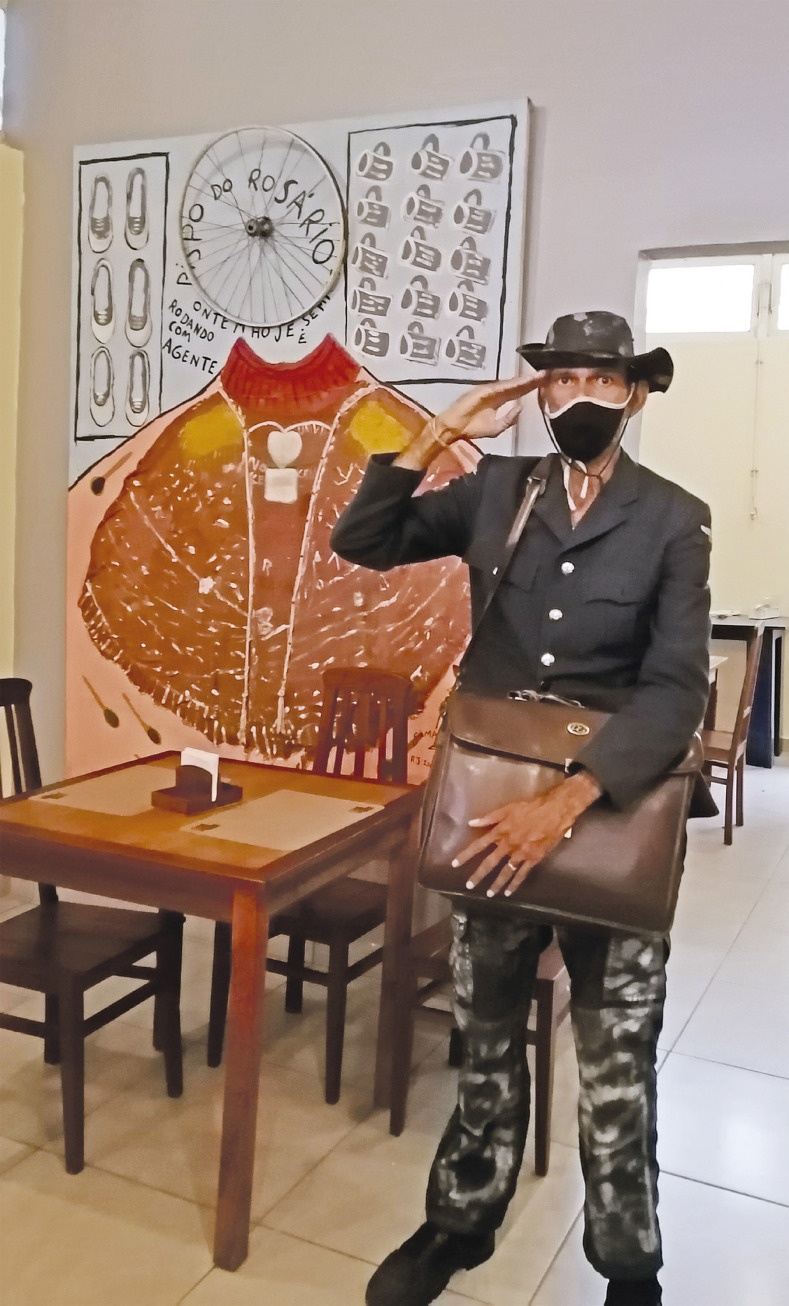
: Gilmar Ferreira no Bistrô do Bispo, no MBRAC (foto feita por Andrea Pires, 2023)

Na análise da obra intitulada *Bispo do Rosário ontem, hoje e sempre rodando com a gente* (Figura 2), Ferreira (2004) destaca o diálogo estabelecido com cinco obras de Arthur Bispo do Rosário. No centro da tela, uma colagem com pintura representa a obra *Manto da apresentação*. No canto superior esquerdo, identificam-se as pinturas de sapatos “Congas” dos pacientes da CJM, fazendo referência à obra *Congas e Havaianas*. Na parte superior direita, pinta canecas da CJM, inspiradas na obra *Canecas*. No centro, na parte superior, o aro de alumínio de uma bicicleta evoca a obra *Roda da fortuna*. Por fim, colheres pintadas ao redor do manto revivem a obra *Talheres*, trazendo memórias relacionadas a essa peça.

Na Figura 2, o artista veste um fardão ou jaqueta e calça pintada por ele mesmo. Em sua pose, ele está com um dos braços levantados, em gesto que se assemelha a uma continência militar, direcionada à fotógrafa. Ao fundo, destaca-se a obra de Ferreira *Bispo do Rosário ontem, hoje e sempre rodando com a gente* (2004), sugerindo uma saudação à memória de Bispo e às suas produções artísticas, com a palavra “ontem” provavelmente evocando a memória de Bispo na extinta CJM. O termo “hoje” remete à imagem de Bispo convivendo com esses artistas; reforçado pelo termo “sempre”, que o vincula aos artistas e usuários, imortalizando, assim, sua presença e sua obra. A presença de Bispo também é evocada na expressão “rodando com a gente”, reiterada pela expressão “a gente”, utilizada de maneira informal para enfatizar que Bispo está presente conosco. Em entrevista realizada em 2023, Ferreira fala sobre a criação dessa obra:

Eu pintei este quadro aqui em homenagem ao Bispo. O nome se fala por bricolagem. O Bispo foi um paciente muito maltratado aqui dentro, ele só não morreu porque no passado ele produzia muita obra. Na época que Bispo estava internado e sendo maltratado ele comia resto de comida. … Colei um aro de bicicleta e o cobertor que eu dormia … é tênis pirulito da época de quando eu era criança … e o bispo tem esse tênis. … o aro quer dizer que Bispo está no céu, mas está rodando com a gente (Ferreira, 2023).

Ferreira e outros artistas, também usuários da CJM, desenvolveram suas produções artísticas no Ateliê Gaia, alcançando reconhecimento como artistas plásticos. As obras de Ferreira têm diferentes destinações, incluindo a venda no Programa de Geração de Trabalho e Renda do MBRAC, baseado em economia solidária. Além disso, ele comercializa sua obra no próprio Ateliê Gaia, em feiras de arte, para colecionadores, em outros museus e *on-line*. O artista também planeja suas produções visando a exposições no MBRAC e em outras instituições no Brasil e no exterior. Ferreira utiliza a renda obtida para melhorar sua qualidade de vida e a de sua família.

Bispo não usufruiu financeiramente de nada do que produziu. Por outro lado, Ferreira tem participado de exposições com suas obras, observando a interação do público com elas. Ele tem a liberdade de interagir com os visitantes, explicar suas obras, vivenciar o *status* de artista e de ver suas criações em espaços dedicados à arte. Bispo não teve essa oportunidade, recebendo apenas poucas pessoas em seu “quarto-forte”. Segundo Wellisch (2006), o mais destacado artista da situação manicomial não teria relacionado seu trabalho com o meio artístico. Ele produzia suas obras para relatar as coisas do mundo, inventando uma técnica singular. Contudo, Bispo alcançou reconhecimento nos últimos anos de vida por meio de documentários, entrevistas e exposições articulados aos movimentos antimanicomiais.

**Figura 3:**
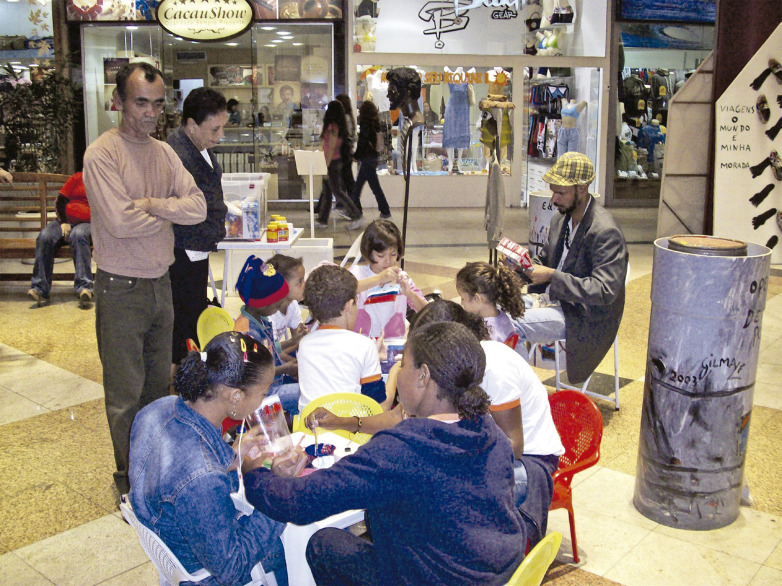
: “Exposição Gilmar Ferreira – Salve a espécie: reciclar é amor”; o artista sentado, de blazer, é Gilmar Ferreira; em pé, de casaco marrom, Arlindo de Oliveira (1951-2024), ambos do Ateliê Gaia (foto feita por Andrea Pires, 2007)

A Figura 3 apresenta a “Exposição Gilmar Ferreira – Salve a espécie: reciclar é amor”. Durante a exposição, o artista interagiu com os visitantes, explicou suas obras e desenvolveu atividades de reciclagem com crianças a partir de suas criações. Seu envolvimento em todas essas etapas da exposição aponta para as mudanças ocorridas no tratamento dos pacientes de saúde mental em relação ao período em que Bispo viveu na CJM.

Em entrevista a um jornal, Ferreira disse: “Eu não pinto porque sou maluco; eu pinto porque sou artista” (Ribeiro, Bastos, 2013). Essa declaração demonstra sua consciência em relação à forma como os outros o percebem. Desse modo, esta pesquisa identificou que as atividades desenvolvidas por Ferreira, como as produções artísticas, a participação nas atividades do MBRAC e em exposições, e a venda das obras, colaboram atualmente para reverter os estigmas associados às doenças mentais (Goffman, 1980; Becker, Arnold, 1986), além de contribuírem para a autonomia e a socialização do artista.

## Considerações finais

Neste estudo, identificamos dois momentos distintos na abordagem terapêutica da saúde mental na CJM. O primeiro abordou as produções artísticas de Arthur Bispo do Rosário, do final da década de 1930 até a década de 1980, produzidas no contexto do modelo asilar de tratamento na CJM. As obras de Bispo podem ser vistas como formas de superação das aflições das clausuras por meio da expressão pessoal, e sua influência se estendeu a outros artistas brasileiros contemporâneos, como Gilmar Ferreira, Jorge Fonseca e José Leonilson Bezerra.

O segundo período corresponde às produções artísticas de Gilmar Ferreira, desde o final da década de 1980 até os dias atuais no IMAS JM, durante o qual ocorreram mudanças significativas nas políticas e na assistência em saúde mental, refletindo as conquistas iniciadas a partir da reforma psiquiátrica brasileira. Esse período também se caracterizou pelo crescimento das atividades do MBRAC e pelo estabelecimento do Ateliê Gaia, que se acredita poder gerar uma mudança no estigma (Goffman, 1980) do usuário da rede de saúde mental.

A produção artística de Gilmar Ferreira no Ateliê Gaia e sua participação nas atividades do MBRAC sugerem momentos de sociabilidade que se contrapõem ao isolamento vivido por Bispo. Ferreira agora desfruta do *status* de artista plástico, desempenhando papel ativo em exposições, na concepção da museografia e em atividades educativas. O reconhecimento de suas criações, que reavivam as imagens das obras de Bispo, demonstra que os usuários de saúde mental podem trilhar novos caminhos. Essas mudanças representam a superação das restrições do passado da CJM, enfatizando a importância das transformações nos tratamentos de saúde mental e a significação dos espaços sociais, como museus, nesse processo.

Embora as descontinuidades históricas entre as condições vividas pelos dois artistas tenham se destacado, não se podem ignorar as continuidades na situação histórica de opressão em que eles se situam. A despeito da quebra da clausura manicomial, evidencia-se a continuidade da inserção na sociedade brasileira, em que os segmentos populares estão condicionados a *habitus* militarizados com os quais se identificam, seja por pragmatismo, seja pela busca de conforto gerado pelas ideias de ordem e hierarquia, características do meio castrense.

Apesar das diferenças entre os processos vivenciados por Bispo e Ferreira, destaca-se um aspecto comum no processo de subjetivação de ambos: a identificação com a disciplina e as práticas associadas ao *ethos* militar. Essa identificação é exemplificada pelo uso de fardas por ambos e pela participação ativa de Bispo em ações de contenção de crises violentas entre os internos, o que lhe valeu a alcunha de xerife. Ferreira, por sua vez, manifestou explicitamente essa identificação quando abordou policiais, afirmando pertencer à milícia. Essa atitude, embora vista negativamente por seus familiares, acabou conduzindo-o a uma nova inserção em situação de tratamento.

Atualmente, existem outros locais que fornecem assistência aos usuários dos serviços de atenção à saúde mental no Rio de Janeiro, como os Caps, onde Ferreira faz seu tratamento, e as residências terapêuticas. Nesse contexto, na CJM, os diversos pavilhões nos quais os pacientes eram internados para tratamento foram sendo desativados ou adaptados para abrigar novas atividades, como clínicas da família, Caps e outros serviços de saúde. Em 27 de outubro de 2022, 98 anos após a fundação da CJM, ocorreu o encerramento das internações psiquiátricas no Núcleo Franco da Rocha, o último núcleo a adotar esse modelo terapêutico. O fato marcou o fim do processo de desinstitucionalização de pacientes psiquiátricos na cidade do Rio de Janeiro, representando um marco na luta antimanicomial e contra o estigma associado à loucura no país.

## Data Availability

Não estão em repositório.

## NOTAS

1 As datas de nascimento de Bispo variam: no registro da Marinha consta 1909, e no da Light and Power, 1911 (Hidalgo, 2011). No prontuário da CJM não há registro da data.

2 O termo usuário foi introduzido pela legislação do SUS (leis n.8.080/1990 e n.8.142/1990) para destacar o protagonismo do que antes era apenas um “paciente” (Amarante, 2007).

3 No Brasil, o termo lepra foi substituído por hanseníase em 1976 dadas as conotações segregadoras. Desde então, o primeiro não é usado em documentos oficiais. Mais informações ver História… (2011).

4 O doutor Juliano Moreira, diretor do Hospital Nacional de Alienados de 1903 a 1930, implementou mudanças significativas em resposta a denúncias de violência e maus-tratos. Durante seus anos à frente do hospital, Juliano Moreira desempenhou papel fundamental na promoção da psiquiatria científica brasileira, tanto no cenário nacional quanto internacional. Sua gestão coincidiu com o período de saneamento e urbanização do Rio de Janeiro sob o mandato do prefeito Pereira Passos (1902-1906) (Venancio, 2005).

5
*Assemblage* é o termo que descreve obras de arte feitas pela combinação de materiais encontrados ou já existentes. Os materiais podem ser escolhidos apenas pelas suas propriedades formais, mas muitas vezes fornecem associações ou alusões. Geralmente se refere a objetos tridimensionais ou construções em relevo (Morgan, 2018).
